# Adrenal Suppression From Mucosal Absorption of Dexamethasone Mouthwash

**DOI:** 10.7759/cureus.113538

**Published:** 2026-07-28

**Authors:** Scarlett Decamps, Jodi B Nagelberg, Kay T Yeung, Karen C McCowen

**Affiliations:** 1 Endocrinology, University of California San Diego, La Jolla, USA; 2 Oncology, University of California San Diego, La Jolla, USA

**Keywords:** adrenal insufficiency, breast cancer, dexamethasone, glucocorticoids, immunotherapy

## Abstract

We present an unusual cause of reversible secondary adrenal insufficiency in a breast cancer patient receiving immunotherapy: systemic absorption of a dexamethasone mouthwash used to “swish and spit,” without being swallowed, which resulted in elevated serum dexamethasone concentrations. Most such patients are presumed to have permanent adrenal insufficiency - well recognized as a complication of immunotherapy - and committed to life-long exogenous glucocorticoid treatment. Recognition of the potential for such oral mucosal steroids to be systemically absorbed, which might be more common than recognized, is critical for the avoidance of inappropriate management in immunotherapy patients.

## Introduction

Chronic exogenous use of glucocorticoids is the most common cause of reversible secondary adrenal insufficiency (AI) [1]. Hypothalamic-pituitary-adrenal (HPA) axis suppression due to external steroid exposure results in low adrenocorticotropic hormone (ACTH), eventual atrophy of the zona fasciculata of the adrenal cortex, and subsequent decreased cortisol production. Recognition of secondary AI is of paramount importance, as abrupt cessation of exogenous steroids may precipitate symptomatic cortisol deficiency [2]. Sources of such exogenous glucocorticoids are widespread and include intra-articular, topical, epidural, and intra-vaginal, and such exposures, while well-described, may be difficult to recognize. HPA suppression due to oral mucosal absorption of non-swallowed steroids has not been systematically investigated, however.

Immunotherapy is an increasingly recognized cause of either primary or secondary AI, most often secondary, through effects on the hypothalamus and pituitary gland [3]. In addition, the effect of immunotherapy tends to be permanent even after discontinuation of the agent. Widespread appreciation of the irreversible nature of AI following immunotherapy often leads to reluctance to taper any glucocorticoid replacement therapy, in contrast to exogenous steroid-induced AI, where many protocols exist to assist clinicians in helping patients come off the replacement. Most oncology treatment protocols using immunotherapy targeting CTLA4, PD1, and PD-L1 include regular testing of the HPA axis. In current clinical practice, the demonstration of a low morning cortisol in the absence of the patient receiving obvious exogenous glucocorticoids leads to a diagnosis of AI and a permanent cortisol replacement strategy, which can be very burdensome to this patient group.

Durvalumab, a PD-L1 immune checkpoint inhibitor, is recognized as a potential cause of multiple different endocrinopathies [4]. Notably, AI has been reported to occur in 0.5% of patients when used as a single agent, with a median onset time of 40 days [5]. We present a case of an immunotherapy-receiving patient who presented with biochemical evidence of reversible AI after using a dexamethasone oral solution (swish and spit) as prophylaxis for mucositis.

This article was previously presented as a meeting abstract at the 2023 Endocrine Society on June 16, 2023.

## Case presentation

A 52-year-old woman with stage IIb triple-negative breast cancer receiving neoadjuvant Datopotamab, Deruxtecan, and Durvalumab was evaluated for AI. Serum cortisol levels were in the normal range (see Table 1) prior to treatment initiation. She had received a single dose of dexamethasone 10 mg IV with her first cycle, but not thereafter. As part of the immunotherapy protocol, serial serum cortisol concentrations were obtained, and two weeks after the baseline, the serum cortisol levels had decreased significantly to very low values. The patient remained without any symptoms of AI. Blood pressure and pulse were normal, and no weight loss had occurred. At no point were glucocorticoids replaced, but one week later, serum cortisol level remained low with concurrently low ACTH. Consideration was given to initiation of hydrocortisone therapy; however, it was deferred per shared decision-making between the patient and her physicians, acknowledging how well she was feeling. 

**Table 1:** Serial Laboratory Evaluation of Adrenal Function Serial measurements of serum cortisol, ACTH, and dexamethasone levels during and after immunotherapy. Laboratory values are presented alongside reference ranges and interpreted qualitatively as low, normal, or high. Trends demonstrate transient suppression of cortisol levels in the setting of elevated dexamethasone concentrations, with recovery following discontinuation. Dashes show times when the analyte was not measured. ACTH, adrenocorticotropic hormone

Time point/clinical context	Cortisol (mcg/dL)	ACTH (pg/mL)	Dexamethasone (ng/dL)	Interpretation (relative to normal)
Baseline (pre-therapy, 11 AM)	7.9	-	-	Cortisol: likely normal
2 weeks after therapy (noon)	1.0	-	-	Cortisol: low
3 weeks after baseline (11 AM)	0.6	5	-	Cortisol: low; ACTH: low
4 weeks after baseline (7 AM)	3.5	18.7	108.2	Cortisol: low; ACTH: normal; dexamethasone: high
7 weeks after baseline (7 AM)	3.3	-	84.3	Cortisol: low; dexamethasone: high
12 weeks after baseline (post-therapy) (9 AM)	9.5	-	-	Cortisol: normal
Reference range	Cortisol (AM) 6.0-18.4 at 6-10 AM	ACTH: ~10-60	Dexamethasone: <50	-

Immunotherapy-induced AI was strongly suspected, and endocrinology consultation was expedited to initiate and monitor glucocorticoid replacement therapy. Her only glucocorticoid exposure had been a dexamethasone oral mouth rinse (swish 10 mL for one minute and spit out, four times daily). Morning cortisol levels one week later were persistently low with inappropriately normal ACTH levels and an elevated dexamethasone level. Dexamethasone concentrations were assessed in the morning, before the first use of the mouthwash, by liquid chromatography-mass spectrometry (LC-MS). Anticipated concentrations following a 1 mg dexamethasone tablet eight hours before a blood level would be 140-295 ng/dL. The technique of swish and spit was reviewed with the patient, and it was confirmed that she was not improperly ingesting the medication. An observational approach was pursued at this time, given hemodynamic stability; dexamethasone swish and spit treatment was continued. The patient was counseled about the signs and symptoms of AI, and instructions as to management under stress were provided. Seven weeks later, repeat labs showed stably low cortisol levels and an elevated dexamethasone level.

Of note, there were no clinical signs suggestive of glucocorticoid exposure or cushingoid features, which were specifically and regularly assessed during this period. The patient understood that there was a risk of AI for at least six months after the initial HPA axis suppression and remained vigilant and in close contact with her physicians. Other pituitary testing revealed normal thyroid function (thyroid-stimulating hormone (TSH) and free T4) and appropriately elevated FSH given post-menopausal status. 

After completing her therapeutic course of treatment, the swish and spit dexamethasone was discontinued. She remained asymptomatic, and repeat labs at 12 weeks after baseline demonstrated an improving cortisol level in the low normal range. In the absence of other sources, low serum cortisol with elevated dexamethasone levels was attributed to dexamethasone swish-and-spit use rather than permanent immunotherapy-related HPA axis suppression.

## Discussion

There is limited literature on dexamethasone swish and spit being absorbed systemically, possibly leading to reversible HPA suppression. The oral mucosa represents an area of enhanced absorption due to its rich vascularity and thin epithelium. Further, there is a greater risk for absorption in inflamed mucosa with impaired barrier function [1]. A typical dexamethasone mouthwash regimen of 0.5 mg/5 mL swished four times daily delivers a total of 4 mg of dexamethasone to the oral cavity per day. Even if only a fraction is absorbed, this could approach or exceed the physiologic daily dose equivalent of 0.25-0.5 mg dexamethasone [2]. However, detailed pharmacokinetic studies of systemic dexamethasone concentrations have not been performed in cancer patients using this mouthwash. Datopotamab has been associated with significant stomatitis, potentially preventing drug continuation; however, steroid swish and spit therapies have substantially lowered the incidence and severity of stomatitis following mTOR inhibitors, leading to such prophylactic strategies being used more broadly [6]. 

A case report published subsequent to our initial presentation described a similar patient using a dexamethasone swish and spit protocol, who developed clinical shock in association with very low serum concentrations of ACTH and cortisol; however, no dexamethasone blood concentration was obtained, and the authors do not report attempting to taper off the glucocorticoid replacement as time went on. It therefore appears likely that this patient might indeed have had true central AI, not definitively caused by exposure to dexamethasone [7]. Given the uncertainties of single-patient case reports, we cannot confirm absolutely that our own case was caused by systemic absorption of the dexamethasone mouthwash. 

Our patient had received a single IV dose of dexamethasone 10 mg, two weeks before the first suppressed cortisol measurement. Previous studies suggest that the duration of cortisol suppression in healthy volunteers is generally less than 48 hours, with maximal suppression noted at 24 hours after the dose [8]. Thus, we are confident that the serially suppressed serum cortisol concentrations that we documented were unrelated to this initial treatment. In addition, we have no way to determine the frequency with which such HPA axis suppression might occur after such an exposure.

At no point was an ACTH stimulation test considered important or performed, as primary AI is an extremely rare side effect of immunotherapy, and there were no risk factors for other etiologies of primary adrenal failure. Often, cortisol values following stimulation with ACTH will be elevated and normal in early secondary AI, so neither a normal nor an abnormal result would have altered our plan for extreme clinical vigilance with our patient. 

Exposure of vaginal mucosa to a variety of glucocorticoid-containing creams for the treatment of inflammatory disorders, such as lichen sclerosis, has demonstrated convincingly that absorption of supposed topical-only therapies can occur (resulting in cases of iatrogenic Cushing syndrome) [9]. In the absence of a Cushingoid phenotype, causality can be difficult to prove. In our case, a commercial lab test exists to measure dexamethasone serum concentrations, which proved highly valuable in confirming our hypothesis. However, in an oncology patient also receiving immunotherapy who has had some exposure to exogenous glucocorticoids other than dexamethasone, the default would be to assume that AI due to immunotherapy had occurred, when no easily available lab test exists to measure that steroid.

We suggest a structured clinical approach and recommend that oncologists: A) perform a meticulous review of all topical, inhaled, intra-articular, and mucosal steroid exposures before diagnosing permanent AI; B) consider transitioning to non-absorbable or non-steroidal prophylactic mouthwashes if HPA axis suppression is detected; and C) consider urgent consultation with endocrinology to assist with evaluation and patient education regarding adrenal crisis and "stress-dosing" of glucocorticoids. 

## Conclusions

This case will raise awareness of non-ingested oral steroid solutions as a reversible cause for consideration when evaluating the etiology of secondary AI. In addition, this patient history emphasizes the need for experienced and thoughtful physicians to use clinical judgment in addition to laboratory testing. Without this approach, patients can be forced into a lifetime of treatment for iatrogenic AI with all its risks of adrenal crisis and exposure to an excess cortisol burden and those side effects.
